# Sex and gender differences in autism spectrum disorder: summarizing evidence gaps and identifying emerging areas of priority

**DOI:** 10.1186/s13229-015-0019-y

**Published:** 2015-06-13

**Authors:** Alycia K Halladay, Somer Bishop, John N Constantino, Amy M Daniels, Katheen Koenig, Kate Palmer, Daniel Messinger, Kevin Pelphrey, Stephan J Sanders, Alison Tepper Singer, Julie Lounds Taylor, Peter Szatmari

**Affiliations:** Autism Science Foundation, 28 W 39th Street #502, New York, NY 10018 USA; Department of Pharmacology and Toxicology, Rutgers University, 41B Gordon Road, Piscataway, 08854 New Brunswick, NJ USA; Department of Psychiatry, University of California San Francisco, 401 Parnassus Ave, LangPorter, 94143 San Francisco, CA USA; William Greenleaf Eliot Division of Child Psychiatry, Washington University School of Medicine, 660 South Euclid Avenue, Campus Box 8134, 63110 St. Louis, MO USA; Autism Speaks, 1 E 33rd St 4th Floor, New York, NY 10016 USA; Initiative for Girls and Women with Autism Spectrum Disorders, Yale Child Study Center, PO Box 207900, 230 South Frontage Road, New Haven, CT 06520-7900 USA; Global and Regional Asperger, Syndrome Partnership, Inc., 419 Lafayette Street, New York, NY 10003 USA; Department of Psychology and Pediatrics, University of Miami, Flipse Building, P.O. Box 249229, Coral Gables, FL 33124-0751 USA; Child Neuroscience Laboratory, Yale Child Study Center, PO Box 207900, 230 South Frontage Road, New Haven, CT 06520-7900 USA; UCSF School of Medicine, Psychiatry, 1550 4th St Bldg 19B, San Francisco, CA 94158 USA; Pediatrics and Special Education, Vanderbilt Kennedy Center Investigator, PMB 40-230 Appleton Pl., Nashville, TN 37203 USA; The Hospital for Sick Children and Centre for Addiction and Mental Health, Centre for Addiction and Mental Health, University of Toronto, 1001 Queen Street West, Toronto, ON M6J 1H4 Canada; Division of Child and Adolescent Psychiatry, Centre for Addiction and Mental Health, University of Toronto, 1001 Queen Street West, Toronto, ON M6J 1H4 Canada

**Keywords:** Female, Autism, Diagnosis, Protection, Symposium, Research

## Abstract

One of the most consistent findings in autism spectrum disorder (ASD) research is a higher rate of ASD diagnosis in males than females. Despite this, remarkably little research has focused on the reasons for this disparity. Better understanding of this sex difference could lead to major advancements in the prevention or treatment of ASD in both males and females. In October of 2014, Autism Speaks and the Autism Science Foundation co-organized a meeting that brought together almost 60 clinicians, researchers, parents, and self-identified autistic individuals. Discussion at the meeting is summarized here with recommendations on directions of future research endeavors.

**Electronic supplementary material:**

The online version of this article (doi:10.1186/s13229-015-0019-y) contains supplementary material, which is available to authorized users.

## Introduction

There has been effort in the last few years to gain attention into the effects of gender on autism prevalence and symptomatology. While the 4:1 sex difference was once the most replicated finding in autism, recent studies have suggested that a number of biases may influence this ratio. Autism Speaks and the Autism Science Foundation co-sponsored a meeting to address basic questions relevant to understanding sex differences in ASD. These questions were identified in collaboration with a steering committee, focusing on issues that could be addressed by scientific experimentation but including those dealing with issues across the lifespan of importance to females with ASD [1]. They are listed by each topic header. A full meeting agenda can be found as Additional file 1. These topics also coincided with a recent thorough analysis of existing knowledge and emerging themes in the literature [2]. The authors recognize that there are more comprehensive reviews of the existing literature on a broader range of topics than has been described in this article. The goal of this short report is to communicate the research priorities highlighted through this meeting, which can inform funding priorities, scientific discoveries, and clinical practice.

## Main text

### Is there a true sex difference in ASD prevalence?

The frequently stated 4:1 ratio is based on an average from multiple studies, both within the US and internationally [3-5]. A male preponderance is not unique to ASD, however, as studies have reliably documented greater prevalence of attention deficit/hyperactivity disorder [6] and other developmental conditions in males compared to females [7]. Some variability in the ratio can be attributed to differences in ascertainment procedures, as the estimates range widely, from 2:1 to 7:1 [5,8,9]. The interaction with intellectual quotient (IQ) also contributes to this variability, with a lower estimate of sex bias observed in cohorts with a lower mean IQ than in ‘high functioning’ cohorts with a higher IQ [10,11]. This interaction with IQ may be compounded by a lower mean IQ in the girls with ASD that are identified in scientific research studies compared to the males [10,8], further exaggerating the sex bias. Finally, there is evidence from studies following younger siblings of individuals with ASD that show that diagnostic biases may lead to an overestimation of sex bias, especially in the high functioning group [10,12]. Collectively, this evidence suggests that at least some of the stated difference in prevalence between males and females may be due to diagnostic/ascertainment differences. Early and accurate diagnosis was termed an ‘essential need’ by self-advocates. Despite variability in ascertainment, a sex difference in ASD prevalence remains with a magnitude of ***at least*** 2:1–3:1, indicating that there is a biological question of sexual dimorphism and ASD symptomatology to be addressed [12-14].

### What is causing the sex difference in ASD prevalence?

One theory, discussed at length at the meeting, proposes that females with ASD are protected against some of the symptoms of ASD (often called the ‘female protective effect’ or FPE) [15]. Under this theory, a higher rate of ASD risk factors should be observed in the average affected female compared to their affected male counterparts; these additional risk factors are required for the female to surpass the higher clinical or diagnostic threshold imparted by the FPE. The FPE has been attributed in other disorders with a strong sex bias, including clubfoot [16]. In support of this theory in ASD, genetic analyses of ASD cohorts have revealed a higher burden of de novo copy number variations (CNVs) [17,18] and *de novo* loss of function point mutations [19,20] in females with ASD than males with ASD. Furthermore, inherited small CNVs are transmitted more frequently from unaffected mothers than unaffected fathers [21].

Epidemiological studies can also address this FPE hypothesis. Since affected females are hypothesized to have a higher average burden of ASD risk factors, it follows that, for inherited risk factors, the parents will also have an increased average burden. This risk has the potential to be transmitted to siblings, in turn increasing their ASD burden, and consequently the rate of sibling ASD recurrence. Therefore, under the FPE model, a higher rate of ASD recurrence is expected in the siblings of affected females than the siblings of affected males; this expectation is often called the Carter Effect. The evidence for such an effect is inconsistent in the ASD literature despite affected population sizes being in the thousands. For ASD diagnosis, no such increase in sibling recurrence rate is observed in the presence of an affected female [9,22,23]. Conversely, considering families with a child that scored highly for subclinical ASD traits, higher scores are observed in siblings if the initial child was female rather than male [15].

### How are females with ASD different than males with ASD?

While the literature has some disparities in identifying clear differences between males and females with ASD, some clear themes have emerged. As mentioned previously, females with a clinical diagnosis of ASD tend to be underrepresented at the higher ends of the IQ distribution [8,24-26]. In addition, females with an ASD diagnosis and IQ scores within the average range show increased functional social behavior compared to males with ASD [27,28]. Females with ASD also show less repetitive behaviors compared to males [12,29], however a stakeholder pointed out that the quality of these repetitive behaviors may be different. For example, a young woman with autism noted that carrying several well-worn books everywhere she goes, and constantly reading them to the detriment of all other social interactions, may be a repetitive behavior that goes undetected. Clinicians noted that circumscribed interests around dolls or babies in females might be misinterpreted as pretend play. In the future, clinicians should be encouraged to consider all behaviors/characteristics of females as a whole and look for repetitive behaviors of any kind, even seemingly benign ones. It is also important for clinicians to obtain as much experience observing both males and females with ASD, so that symptoms are correctly identified.

These group level differences suggest that females may be under diagnosed because of differing symptom presentation. As suggested previously (for review see [30]), males may show more of the behaviors that trigger a clinical evaluation, such as hyperactivity and aggression. If females with an ASD diagnosis and IQ scores above 70 are perceived by clinicians as being more social, their presentation of symptoms may be misinterpreted and accurate diagnosis may be delayed. In addition, it is possible that sex-specific characteristics in typically developing males and females may mask some of the core deficits of ASD. Females with ASD and high IQ also tend to have higher language ability, possibly reflecting the sex difference in language in typically developing males and females [28,31]. Other examples of sexually dimorphic differences are memory, cognitive flexibility, verbal fluency, and social-communication [12,28,32,33]. Adding on a layer of complication is differential expectations for females vs. males among parents and clinicians with respect to social-communication and play behaviors [34]. These factors clearly may impact prevalence numbers in males and females.

These inherent sex differences between males and females without ASD should be seen as different baselines to draw comparisons. However, in the field of ASD research, females without ASD are rarely compared to females with ASD, and the influence of being female is not studied. Therefore, the M:F prevalence differences may be inflated due to biological and sociological differences not specific to ASD. For example, this was recently shown to be the case in the frequency of genetic mutation [35]. Non-biological factors that may influence diagnosis deserve a more thoughtful and comprehensive discussion than time allowed at this meeting.

### When do differences emerge in males and females with ASD?

Although past research has also documented gender differences in age at diagnosis [36-39]. The differences in symptoms in males and females are not apparent in toddlerhood. Recently published data [13,40] reported that toddler males and females with ASD do not show any differences in behavioral features, suggesting that the differences in symptoms do not show up until later in development. Examining three year olds with an older sibling with ASD reveals males are overrepresented in clusters of three year olds with symptoms [9,14]. For example, in non-diagnosed siblings, differences in symptom presentation were seen regardless of risk group, with males showing higher ASD severity scores and lower verbal functioning [14], indicating a protection of females to autism-related deficits. It is important to note that social interaction skills in males and females differ across development [41], which may explain lack of presentation in toddlers. In fact, recently, a retrospective parent interview revealed qualitative differences in social behaviors and interests in both preschool males and females with autism. This includes use of complex imitation and circumscribed interests in things like dolls and feathers, rather than parts of toys [42]. It is also possible that symptoms may not emerge until social pressures change in adolescence. This issue emphasizes the need to better study trajectories of development rather than individual cross sectional studies. Specifically, longitudinal investigations will allow researchers to examine whether ASD symptoms emerge more quickly or more gradually in males or females.

### What are the unique challenges to females with ASD as they transition into adulthood?

The stakeholder community pointed out the gap in understanding of factors that increase quality of life, productivity, and the underlying factors impeding those issues. They voiced that the only way to achieve that is to spend a lot of time, in a variety of diverse situations, with people with ASD. This may include a range of settings like naturalistic social situations and workplace interactions. This is essential to better understanding priorities of the ASD community. The issue of employment was discussed at the meeting, as it is an area that has been studied scientifically. Existing data are limited, but suggest that females with ASD are able to obtain, but not maintain, employment or post-secondary education [43]. The reasons are still being explored, but may include expectations by employers, other staff, and the male bias in the type of skills offered in job training. More data are needed on this topic and others, including job coaching, and job training. Efforts in the US and in Europe (www.autisminpink.net) are providing services for females with ASD, but data on the effectiveness of different services, treatments and training in females needs to be gathered as well. There are remarkably few studies on relationships of females with ASD (e.g., romantic or sexual relationships) beyond relatively minimal measures of friendship. However, scientific studies are illustrating the need for gender specific strategies for domains like social skills training [44].

## Conclusions

Accurate and early diagnosis of autism in both sexes is essential, not only for understanding sex differences in ASD, but also for providing appropriate resources and services. Accurate diagnosis and identification of autism-like features is also necessary for lifelong support of women whose impairments may be traditionally under recognized. These advancements will require further research and scientific study. While not intended to be a comprehensive list, research priorities, challenges recommendations and impact of these recommendations that emerged from the discussion at the October meeting are provided in Table 1.

**Table 1:** **Comprehensive list of research priorities, challenges, recommendations, and implications of recommendations of research**

**Research priority**	**Challenges**	**Recommendation(s)**	**Implications**
Better identification and diagnosis of females	Diagnostic norms developed in adolescent males	Clinical guidelines or recommendations for clinicians, encouragement of clinicians to observe both males and females with ASD in training	Changes in M:F bias in prevalence
	Societal and cultural expectations of males and females	Provide comparisons between ASD females and typical females across studies	Understanding of specific needs of females with ASD
	Compensatory mechanisms in social behaviors in females, masking symptoms and hiding diagnosis	Reduced reliance on clinical samples for data collection	Improvement in services and resources available for females with ASD
	Qualitative differences in symptoms between males and females in development	Examination of early signs and symptoms, including trajectories in at risk infants.	Earlier detection of ASD in females
Characterization of male: female differences in core and associated symptoms	Low sample sizes of females enrolled in research studies	Data sharing, pooling, repository efforts	Improved representation of females in ASD research and specific recommendations for females with ASD
	Restriction of signs and symptoms to ASD diagnosis	Including ASD associated symptoms, broader phenotype, and understanding of heterogeneity	Potential sex specific diagnostic criteria
Biological differences between males and females	Variability introduced with inclusion of females in research	Sex included as a covariate in research studies, especially animal models	Identification of protective mechanisms for translational impact.
	Limited understanding of human sexual dimorphism at a molecular, cellular or anatomical level	Basic science focused specifically on human sexual dimorphism	Understanding role of male/female physiological differences in protection of some ASD symptoms

## Additional file

Additional file 1:
**Meeting Agenda, Agenda including presenter information for the Sex and Gender Differences in Autism meeting, October 29, 2014.**

## Abbreviations

ASD: Autism spectrum disorder
CNV: Copy number variation
FPE: Female protective effect
IQ: Intellectual quotient
