# Supplementary material for: Sex and gender differences in autism spectrum disorder: summarizing evidence gaps and identifying emerging areas of priority
Source: Mol Autism. 2015 Jun 13;6:36. doi: 10.1186/s13229-015-0019-y (PMC4465158; doi:10.1186/s13229-015-0019-y)
Supplement: Additional file 1: — Meeting Agenda, Agenda including presenter information for the Sex and Gender Differences in Autism meeting, October 29, 2014. [file 13229_2015_19_MOESM1_ESM.pdf]

AUTISM SPEAKS™ It's time to listen.

AUTISM SCIENCE FOUNDATION

AGENDA

Sex and Gender Differences in Autism Spectrum Disorders

October 29, 2014
Doubletree Metropolitan Hotel, NYC

**8:30 am – 9:00 am**	**Continental breakfast**	
**9:00 am – 9:30 am**	**Welcome and Introductions**	
**9:30 am – 9:45 am**	**Perspectives of a parent and women on the spectrum**	Alison Singer, Kate Palmer
**9:45 am – 10:15am**	**The big question: What are the current challenges in understanding sex differences in ASD?**	Presenter: Peter Szatmari<br>Facilitator: Meng-Chuan Lai<br>Discussants: Amy Daniels, Alice Kau, Alice Luo Clayton, Lisa Gillotty, Kate Palmer, Avi Reichenberg
**10:15 am – 10:45 am**	Discussion	
**10:45 am -11:00 am**	**Break**	
**11:00 am – 11:30 am**	**What do girls and women "look" like and are they underdiagnosed?**	Presenter: Somer Bishop<br>Facilitator: Lonnie Zwaigenbaum<br>Discussants: Audrey Thurm, Meghan Miller, Becky Landa, Rene Jamison, Pamela Ventola
**11:30 am – 12:00 pm**	Discussion	
**12:00 pm – 1:30 pm**	**Lunch**<br>**Each table will be asked to pick from the following questions to discuss:**<br>• **What is the main challenge of women vs. men with ASD?**<br>• **What is not being researched but should be?**<br>• **If researchers are missing girls to study, what can be done to find them?**	

Text

**1:30 pm – 2:00 pm**	**What are the early developmental trajectories of boys and girls? When do we see the differences?**	Presenter: Daniel Messinger<br>Facilitator: Ami Klin<br>Discussants: Helen Tager-Flusberg, Sara Webb, Greg Young, Kasia Chawarska, Ted Hutman
**2:00 pm – 2:30 pm**	Discussion	
**2:30 pm – 3:00 pm**	**What happens to girls with autism in adolescence and adulthood? What are the gender issues that affect outcome and how do we study them?**	Presenter: Julie Taylor<br>Facilitator: Kathy Koenig<br>Discussants: Marisela Huerta, Jessica Schuttler, Sharon Valencia, Carmen McLean
**3:00 pm – 3:30 pm**	**Discussion**	
**3:30 pm – 4:00 pm**	**What are the causes of the disparity in boys and girls? Are they protected in some way?**	Presenter: Stephan Sanders<br>Facilitator: John Constantino<br>Discussants: Christine Nordahl, Kevin Pelphrey, Donald Pfaff, Sara Schaafsma, Alison Jack
**4:00 pm – 4:30 pm**	Discussion	
**4:30 pm – 5:00 pm**	**Priorities going forward and future plans for research**	Facilitator: Alycia Halladay

Questions can be emailed to ahalladay@autismsciencefoundation.org
or texted to 908 963 5345
